# Competence of *Aedes aegypti*, *Ae. albopictus*, and *Culex quinquefasciatus* Mosquitoes as Zika Virus Vectors, China

**DOI:** 10.3201/eid2307.161528

**Published:** 2017-07

**Authors:** Zhuanzhuan Liu, Tengfei Zhou, Zetian Lai, Zhenhong Zhang, Zhirong Jia, Guofa Zhou, Tricia Williams, Jiabao Xu, Jinbao Gu, Xiaohong Zhou, Lifeng Lin, Guiyun Yan, Xiao-Guang Chen

**Affiliations:** Southern Medical University, Guangzhou, China (Z. Liu, T. Zhou, Z. Lai, Z. Zhang, Z. Jia, T. Williams, J. Xu, J. Gu, X. Zhou, X.-G. Chen);; University of California, Irvine, California, USA (G. Zhou, G. Yan);; Guangdong Provincial Center for Disease Control and Prevention, Guangzhou (L. Lin)

**Keywords:** *Aedes aegypti*, *Aedes albopictus*, *Culex quinquefasciatus*, vector competence, Zika virus, mosquitoes, viruses, vector-borne infections, China

## Abstract

In China, the prevention and control of Zika virus disease has been a public health threat since the first imported case was reported in February 2016. To determine the vector competence of potential vector mosquito species, we experimentally infected *Aedes aegypti, Ae. albopictus*, and *Culex quinquefasciatus* mosquitoes and determined infection rates, dissemination rates, and transmission rates. We found the highest vector competence for the imported Zika virus in *Ae. aegypti* mosquitoes, some susceptibility of *Ae. albopictus* mosquitoes, but no transmission ability for *Cx*. *quinquefasciatus* mosquitoes. Considering that, in China, *Ae. albopictus* mosquitoes are widely distributed but *Ae. aegypti* mosquito distribution is limited, *Ae. albopictus* mosquitoes are a potential primary vector for Zika virus and should be targeted in vector control strategies.

Zika virus is a mosquitoborne flavivirus that poses a serious threat worldwide (*1*). Because cases of Zika virus disease in humans have been sporadic and symptoms mild, Zika virus has been neglected since its discovery in 1947 (*2**,**3*). The first major Zika virus outbreak was reported on Yap Island in Micronesia in 2007 (*4*). However, the Zika virus disease outbreak in French Polynesia during 2012–2014 surprised the public health communities because of the high prevalence of Guillain-Barré syndrome (*5*). In addition, the ongoing Zika virus epidemic in the Americas since 2015 was associated with congenital infection and an unprecedented number of infants born with microcephaly (*6**,**7*). In 2015, the Zika virus epidemic spread from Brazil to 60 other countries and territories; active local virus transmission (*8*) and cases of imported Zika virus disease are occurring all over the world (*9**,**10*). In view of the seriousness of the epidemic, the World Health Organization declared the clusters of microcephaly and Guillian-Barré syndrome a Public Health Emergency of International Concern (*11*).

Experimental studies have confirmed that *Aedes* mosquitoes, including *Ae. aegypti*, *Ae. albopictus*, *Ae. vittatus*, and *Ae. luteocephalus*, serve as vectors of Zika virus (*12**–**15**)*. However, vector competence (ability for infection, dissemination, and transmission of virus) differs among mosquitoes of different species and among virus strains. *Ae. aegypti* mosquitoes collected from Singapore are susceptible and could potentially transmit Zika virus after 5 days of infection; however, no Zika virus genome has been detected in saliva of *Ae. aegypti* mosquitoes in Senegal after 15 days of infection (*12**,**14*). *Ae. albopictus* mosquitoes are a secondary vector for Zika virus transmission (*16*). In Italy, the population transmission rate is lower and the extrinsic incubation period is longer in *Ae. albopictus* than in *Ae. aegypti* mosquitoes (*17*). Transmission of Zika virus may also involve mosquitoes of other species such as those of the genera *Anopheles* and *Culex;* the virus had been detected in *An. coustani* and *Cx. perfuscus* mosquitoes from Senegal (*18**,**19*).

In February 2016, China recorded its first case of Zika virus infection in Jiangxi Province; the case was confirmed to have been caused by virus imported from Venezuela (*20*). Since then, 13 cases caused by imported Zika virus have been reported from several provinces (*21*); no evidence of autochthonous transmission has been found. In China, *Ae. aegypti* mosquitoes are found only in small areas of southern China, including Hainan Province and small portions of Yunnan and Guangdong Provinces (*22*). The predominant mosquitoes across China, especially in cities, are *Ae. albopictus* and *Cx. quinquefasciatus* (*23**,**24*); *Ae. albopictus* mosquitoes are the primary vector of dengue virus (family *Flaviviridae*) (*25*). *Cx. quinquefasciatus* mosquitoes are the primary vector for the causative organisms of St. Louis encephalitis, Rift Valley fever, lymphatic filariasis, and West Nile fever (*26*). The potential for *Cx. pipiens* mosquitoes to be Zika virus vectors (*27*) needs further confirmation. Because cases of Zika virus disease caused by imported virus have been reported in China, we investigated the potential vectors.

## Materials and Methods

### Mosquitoes 

The Guangdong Provincial Center for Disease Control and Prevention collected *Ae*. *albopictus* and *Cx. quinquefasciatus* mosquitoes from different sites in the cities of Foshan (in 1981) and Guangzhou (in 1993) in Guangdong Province. In 2005, the China Center for Disease Control and Prevention collected *Ae. aegypti* mosquitoes from the city of Haikou in Hainan Province. All mosquitoes were maintained under standard insectary conditions of 27 ± 1°C, 70%–80% relative humidity, and a light:dark cycle of 16 h:8 h. To obtain enough individuals for the experiments, we collected eggs from mosquitoes of all 3 species and hatched them in dechlorinated water in stainless steel trays. The larvae (150–200/L water) were reared and fed daily with yeast and turtle food. Pupae were put into 250-mL cups and placed in the microcosm (20 cm × 20 cm × 35 cm cage covered with nylon mesh) until they emerged. Adults were kept in the microcosms and given 10% glucose solution ad libitum.

### Zika Virus

Zika virus (GenBank accession no. KU820899.2), provided by the Guangdong Provincial Center for Disease Control and Prevention, was originally isolated from a patient in China in February 2016 and classified as the Asian lineage (*28**,**29*). The virus had been passaged once via intracranial inoculation of suckling mice and twice in C6/36 cells. In the laboratory at Southern Medical University (Guangzhou, China), C6/36 cells were infected by virus stocks with a multiplicity of infection of 1 and left to grow at 28°C for 5–7 days. The cells were suspended and separated into an aliquot and stored at −80°C. The frozen virus stock (3.28 ± 0.15 log_10_ copies/μL) was passaged once through C6/36 cells before the mosquitoes were infected. The fresh virus suspension (5.45 ± 0.38 log_10_ copies/μL) was used to prepare the blood meal.

### Infection of Mosquitoes

We transferred 5–7-day-old female *Ae. aegypti*, *Ae. albopictus*, and *Cx. quinquefasciatus* mosquitoes to 500-mL cylindrical cardboard containers covered with mesh, where they were starved for 24–48 h. The infectious blood meal was prepared by mixing defibrinated sheep blood (Solarbio, Beijing, China) with fresh virus suspension at a ratio of 1:2. The blood meal was warmed to 37°C and transferred into a Hemotek blood reservoir unit (Discovery Workshops, Lancashire, UK). Mosquitoes were then fed by using the Hemotek blood feeding system. Quantitative reverse transcription PCR (qRT-PCR) was used to detect the virus concentration (copy level) in the blood meal before and after feeding. After 30 min of exposure to the infectious blood meal, mosquitoes were anesthetized with diethyl ether. Fully engorged females were transferred to 250-mL paper cups covered with net (10–15 mosquitoes/cup). The infected mosquitoes were provided with 10% glucose and maintained in an HP400GS incubator (Ruihua, Wuhai, China) at 28°C, 80% relative humidity, and a light:dark cycle of 16 h:8 h. The experiments were conducted according to standard procedures in a Biosafety Level 2 laboratory.

### Zika Virus Infection in Whole Mosquitoes 

To determine Zika virus infections in *Ae*. *aegypti*, *Ae*. *albopictus* and *Cx. quinquefasciatus* mosquitoes, we selected 18–30 mosquitoes at postinfection days (dpi) 0 (same day as blood meal), 4, 7, 10, and 14. Each mosquito was placed in 50 μL TRIzol (Ambion, Life Technologies, Carlsbad, CA, USA) and homogenized in a tissue grinder (Kontes, Vineland, NJ, USA). Total RNA was extracted according to the manufacturer’s protocol of TRIzol reagent and dissolved in 20 μL RNase-free water.

Zika virus cDNA synthesis reaction was performed by using the GoScript Reverse Transcription System (Promega, Madison, WI, USA). Total RNA and 10 μM Zika virus reverse primer (3′ untranslated region: 5′-ACCATTCCATTTTCTGGC-3′) were incubated, and cDNA was synthesized according to the procedure.

The nonstructural protein 1 (NS1) primers of Zika virus were designed by NCBI/Primer-BLAST (https://www.ncbi.nlm.nih.gov/tools/primer-blast/index.cgi?LINK_LOC=BlastHome), which was selective for 296-bp nucleotide (forward: 5′-ACCCAAGTCTTTAGCTGGGC-3′; and reverse: 5′-CTGGTTCTTTCCTGGGCCTT-3′). The following PCR conditions were used: 94°C for 3 min, followed by 35 cycles of 94°C for 30 s, 60°C for 30 s, 72°C for 30 s, and 72°C for 7 min. The PCR products were examined by use of 1% agarose gel electrophoresis. The target fragment was cloned into pMD18-T vector (Takara, Dalian, China) and sequenced.

### Quantification of Zika Virus in Mosquitoes 

The viral genome in the Zika virus–positive mosquitoes was determined by using absolute qRT-PCR. First, we constructed the standard. A 141-bp fragment across capsid and propeptide regions of Zika virus was amplified by specific primers (forward: 5′-GGAGAAG AAGAGACGAGGCG-3′; and reverse: 5′-GATATGGCCTCCCCAGCATC-3′) and cloned into pMD18-T vector. After sequencing, the recombinant plasmid was linearized by *EcoR* I. The concentration of plasmid was detected by NanoDrop 2000 Spectrophotometer (Thermo Scientific, Wilmington, DE, USA). A standard curve (linear curve slope −3.447, Y intercept 38.312, R^2^ 1, amplification efficiency 95.029) was generated from a range of serial 10-fold dilutions of the plasmid (6.23 × 10^2^**–**6.23 × 10^7^ copies/μL).

Each 20 μL of qRT-PCR was amplified by a 7500 Real-Time PCR System (Applied Biosystems, Foster City, CA, USA) under the following conditions: 1 cycle at 50°C for 2 min, 95°C for 2 min; 40 cycles at 95°C for 15 s, 60°C for 15 s, and 72°C for 1 min. Zika virus RNA copies from each sample were quantified by comparing cycle threshold value with the standard curve. The efficiency of this qRT-PCR system was evaluated by using blank control, uninfected C6/36 cells, C6/36 cells infected with Zika virus or DENV, and mosquitoes infected with Zika virus or DENV; the result displayed that its minimum detecting amount is 6.23 copies/μL of Zika virus and that the specificity is 100%.

### Zika Virus Infection in Mosquito Tissues 

To further analyze Zika virus tropisms and vector competence in *Ae. aegypti*, *Ae. albopictus*, and *Cx. quinquefasciatus* mosquitoes, we infected another batch of mosquitoes with Zika virus and then dissected the midgut, head, and salivary glands of each mosquito at dpi 0, 4, 7, 10, and 14 by using 18–30 mosquitoes per time point. The legs and wings of mosquitoes were removed and placed into cold phosphate-buffered saline. Each tissue was dissected and washed 3 times in phosphate-buffered saline and transferred to 50 μL TRIzol (*30*). Following the above-mentioned procedure, we extracted total RNA, and the NS1 region of Zika virus from samples was detected by RT-PCR. The viral RNA copies from the positive samples were quantified by qRT-PCR. For those mosquitoes with Zika virus–negative midguts by RT-PCR and qRT-PCR, which we considered to be uninfected, we did not further analyze the heads and salivary glands. Vector competence of mosquitoes of 3 species was evaluated by calculating infection rate (no. infected midguts/no. tested midguts), dissemination rate (no. infected heads/no. infected midguts), transmission rate (no. infected salivary glands/no. infected midguts), and population transmission rate (no. infected salivary glands/no. tested mosquitoes).

### Statistical Analyses

All statistical analyses were performed by using SPSS version 20.0 (IBM, Chicago, IL, USA). Logistic regression was used to compare the infection, dissemination, and transmission rates for different mosquito species at the same time or for the same species of mosquito at different times. p value significance was corrected by Bonferroni adjustments. The Zika virus RNA copy levels were log-transformed and then compared among mosquitoes of different species at the same time or among mosquitoes of the same species at different times by using post hoc Tukey honest significant difference tests.

## Results

### Zika Virus Infection of and Replication in Mosquitoes

**Figure 1:**
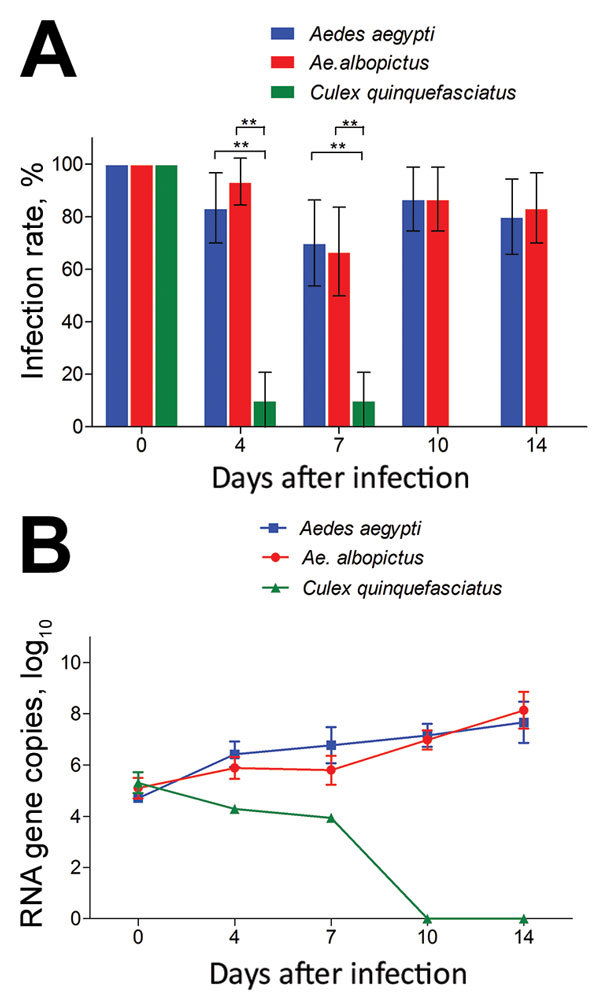
Infection rates and virus reproduction for Zika virus in *Aedes* *aegypti*, *Ae*. *albopictus*, and *Culex* *quinquefasciatus* mosquitoes in China. A) Infection rate. Error bars represent 95% CIs. **, p<0.01. B) Zika virus RNA titers in the whole mosquito bodies was detected by quantitative reverse transcription PCR. The results are expressed as mean ± SD.

After the 414 mosquitoes (138 of each of the 3 species) had ingested the infectious blood meal (dpi 0), RT-PCR indicated that all were Zika virus positive (Figure 1, panel A). The high proportion of mosquitoes infected with Zika virus was observed for *Ae. aegypti* and *Ae. albopictus* mosquitoes at dpi 4, 7, 10, and 14 (Figure 1, panel A). Infection rates were similar for *Ae. aegypti* and *Ae. albopictus* mosquitoes at all time points (z = 1.169, −0.277, 0.0001, 0.333; p>0.05) (Figure 1, panel A). However, at dpi 4 and 7, the infection rate for *Cx. quinquefasciatus* mosquitoes was significantly lower than that for *Ae*. *aegypti* mosquitoes (z = −4.924, −4.186; p<0.01) and *Ae. albopictus* mosquitoes (z = −1.169, −4.007; p<0.01) (Figure 1, panel A). After dpi 7, no Zika virus was detected in the midgut of *Cx. quinquefasciatus* mosquitoes (Figure 1, panel A).

The amount of Zika virus from the mosquitoes with midgut infection was further tested by qRT-PCR. The trend for mean Zika virus copies in *Ae*. *aegypti* and *Ae*. *albopictus* mosquitoes was an increase with time after infection, but that for *Cx. quinquefasciatus* mosquitoes was a decrease (Figure 1, panel B). For *Ae*. *aegypti* mosquitoes, Zika virus copies increased quickly from dpi 0 to 4 (p<0.05 by Tukey honest significant difference test), then increased gradually. For *Ae*. *albopictus* mosquitoes, the trend for copy levels of Zika virus was similar to that for *Ae. aegypti* mosquitoes, but levels were slightly lower before dpi 7 (p<0.05). However, the copy levels were the same for mosquitoes of the 2 species at dpi 10 and 14 (p>0.05). For *Cx. quinquefasciatus* mosquitoes, the virus copy levels were low before dpi 7 and totally diminished afterward (Figure 1, panel B).

### Vector Competence of Mosquitoes after Oral Challenge

**Figure 2:**
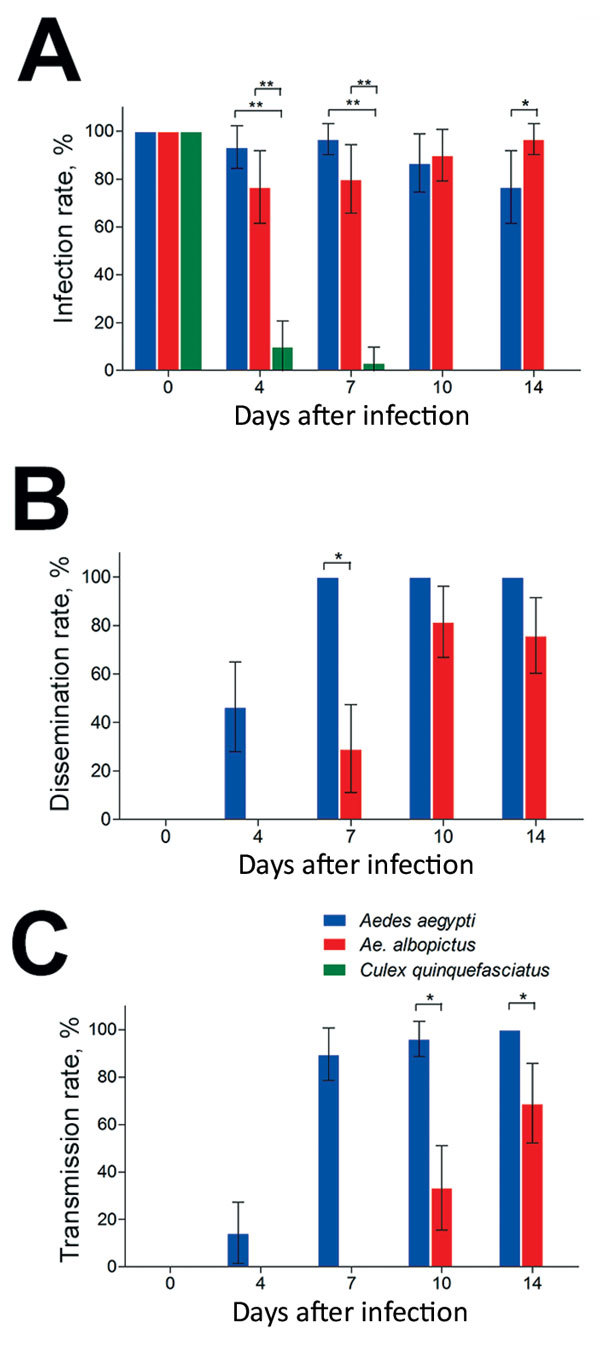
Vector competence of Zika virus in *Aedes aegypti*, *Ae. albopictus*, and *Culex quinquefasciatus* mosquitoes in China. The midguts, heads, and salivary glands from mosquitoes of the 3 species were dissected at 0, 4, 7, 10, and 14 days after infection, and Zika virus was detected by reverse transcription PCR. A) Infection rate (no. positive midguts/total no. midguts). B) Dissemination rate (no. positive heads/no. positive midguts). C) Transmission rate (no. positive salivary glands/no. positive midguts). Error bars indicate 95% CIs. *p<0.05; **p<0.01.

The infection, dissemination, and transmission rates for Zika virus were assessed by detecting infection status of mosquito midguts, heads, and salivary glands. Another 414 mosquitoes (138 from mosquitoes of each species) were infected by Zika virus, and the midguts were measured; the overall infection rates were 89.86% for *Ae. aegypti*, 87.68% for *Ae. albopictus*, and 15.94% for *Cx. quinquefasciatus* mosquitoes (Table). At dpi 0, 100% of midguts were infected because of the undigested blood meal containing the virus, while no virus appeared in other tissues. High infection rates were maintained in *Ae. aegypti* and *Ae. albopictus* mosquitoes during the experimental period; no significant difference between *Ae. aegypti* and *Ae. albopictus* mosquitoes was found at dpi 4, 7, and 10 (z = 1.706, 1.777, 0.401; p>0.05) (Figure 2, panel A). At dpi 14, the infection rate for *Ae. albopictus* was higher than that for *Ae. aegypti* mosquitoes (z = 1.971; p = 0.04873). Compared with the infection rates for *Ae. aegypti* and *Ae. albopictus* mosquitoes, that for *Cx. quinquefasciatus* mosquitoes was significantly lower at dpi 4 (z = −5.081, −4.539; p<0.01) and 7 (z = −4.682, −4.264; p<0.01), and no midguts were positive for Zika virus at dpi 10 and 14 (Figure 2, panel A).

**Table T1:** Rates of Zika virus infection, dissemination, transmission, and population transmission for *Aedes aegypti, Ae. albopictus, and Culex quinquefasciatus* mosquitoes, China

Rate	Mosquito species		
*Ae. aegypti*	*Ae. albopictus*	*Cx. quinquefasciatus*
Infection*	124/138 (89.86)	121/138 (87.68)	22/138 (15.94)
Dissemination†	91/124 (73.39)	51/121 (42.15)	0/22 (0)
Transmission‡	78/124 (62.90)	29/121 (23.97)	0/22 (0)
Population transmission§	78/138 (56.52)	29/138 (21.01)	0/138 (0)

*No. infected midguts/no. tested midguts (%). †No. infected heads/no. infected midguts (%). ‡No. infected salivary glands/no. infected midguts (%). §No. infected salivary glands/no. infected mosquitoes (%).

The dissemination of Zika virus in the heads of *Ae. aegypti* mosquitoes started from dpi 4 and increased rapidly up to 100% after dpi 7 (Figure 2, panel B). The spread of Zika virus in the heads of *Ae. albopictus* mosquitoes was first detected at dpi 7, and the rate was lower than that for *Ae. aegypti* mosquitoes at the same time point (z = −3.832; p<0.05) (Figure 2, panel B). Peak dissemination occurred during dpi 4–7 for *Ae. aegypti* (z = 4.344; p<0.001) and 7–10 for *Ae. albopictus* (z = 3.543; p<0.001) mosquitoes. Overall, Zika virus infection was disseminated in 73.39% of midgut-infected *Ae. aegypti* mosquitoes but only 42.15% of midgut-infected *Ae. albopictus* mosquitoes (Table). Zika virus was not detected in the head tissues of *Cx. quinquefasciatus* mosquitoes.

For *Ae. aegypti* mosquitoes, the detection of Zika virus in salivary glands was consistent with that in heads (Figure 2, panel C). Transmission of Zika virus by *Ae. albopictus* mosquitoes (which began at dpi 10 and increased to 68.97% at dpi 14) was lower at dpi 14 than that for *Ae. aegypti* mosquitoes at the same time (z = −3.561, −2.550; p<0.05) (Figure 2, panel C). A significant difference in transmission was detected during dpi 4–7 (z = 4.847; p<0.001) for *Ae. aegypti* and dpi 10–14 (z = 4.847; p = 0.0116) for *Ae. albopictus* mosquitoes. Zika virus was detected in the salivary glands of 78 (62.90%) midgut-infected *Ae. aegypti* and 29 (23.97%) midgut-infected *Ae. albopictus* mosquitoes. Furthermore, the population transmission rates of Zika virus were 56.52% for *Ae. aegypti*, 21.01% for *Ae. albopictus*, and 0% for *Cx. quinquefasciatus* mosquitoes (Table[Table T1]).

**Figure 3:**
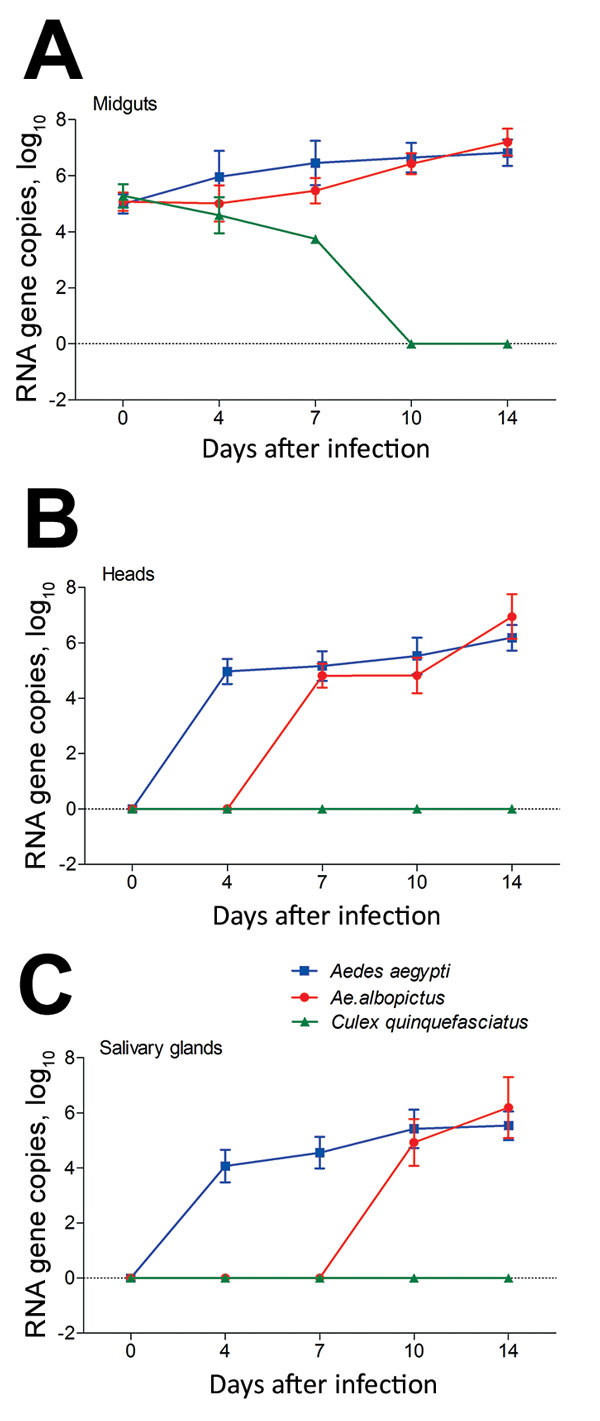
Zika virus RNA copies in infected midguts (A), heads (B), and salivary glands (C) of *Aedes aegypti*, *Ae. albopictus*, and *Culex quinquefasciatus* mosquitoes in China*.* Results are expressed as means ± SD. Dotted lines indicate the level below which minimum value could not fall. Error bars indicate SDs.

The amount of Zika virus in mosquito midguts, heads, and salivary glands was measured by qRT-PCR. The Zika virus copies (log_10_) in midguts of *Ae. aegypti*, *Ae. albopictus*, and *Cx. quinquefasciatus* mosquitoes did not differ significantly at dpi 0 (p>0.05). For *Ae. aegypti* mosquitoes, the Zika virus copies (log_10_) of midguts at dpi 4 were rapidly raised to 5.96 ± 0.92, which was higher than that at dpi 0 (5.00 ± 0.34) (p<0.05). Levels then increased continuously over time and reached 6.82 ± 0.47 at dpi 14 (Figure 3, panel A). For *Ae. albopictus* mosquitoes, the trend of increasing mean Zika virus copies was slow before dpi 7 and significantly lower than that for *Ae. aegypti* at the same time (p<0.05). After that, the growth of Zika virus became rapid and the Zika virus copies (log_10_) at dpi 14 reached 7.20 ± 0.48, which exceeded that in *Ae. aegypti* mosquitoes (p<0.05) (Figure 3, panel A). However, the amount of Zika virus continued to decrease in *Cx. quinquefasciatus* mosquito midguts after infection (Figure 3, panel A).

The number of Zika virus RNA copies (log_10_) in heads of *Ae. aegypti* mosquitoes continually increased from dpi 4 (4.97 ± 0.45) to 14 (6.19 ± 0.46) but remained stable for *Ae. albopictus* mosquitoes from dpi 7 (4.82 ± 0.43) to 10 (4.82 ± 0.64) and then reached 6.95 ± 0.81 at dpi 14 (Figure 3, panel B). At dpi 10, the number of virus copies in *Ae. aegypti* mosquitoes was higher than that in *Ae. albopictus* mosquitoes, whereas levels inverted at dpi 14 (p<0.05) (Figure 3, panel B). The trend of Zika virus in salivary glands was similar to that in the heads of *Ae. aegypti* mosquitoes. Compared with the Zika virus copies (log_10_) at dpi 0 (0), the value (5.54 ± 0.52) was apparently higher at dpi 14 (p<0.05) (Figure 3, panel C). Zika virus was detected in the salivary glands of *Ae. albopictus* mosquitoes during dpi 10–14, and the value at dpi 14 (6.19 ± 1.10) was higher than that at dpi 10 (4.92 ± 0.85). Furthermore, the number of virus copies in *Ae. albopictus* mosquitoes was higher than that in *Ae. aegypti* mosquitoes at dpi 14 (p<0.05) (Figure 3, panel C).

## Discussion

Because of the absence of vaccines and specific treatment, the major approach to prevention and control of Zika virus disease is vector control (*31*). Identification of the mosquito species that could transmit Zika virus and determination of the extrinsic incubation period of Zika virus will provide a guide for vector control. In this study, we demonstrated experimentally that *Ae. aegypti* and *Ae. albopictus* mosquitoes in China possess the ability to transmit Zika virus, whereas *Cx. quinquefasciatus* mosquitoes were not able to transmit the virus under our laboratory conditions.

Our results demonstrate that *Ae. aegypti* mosquitoes could serve as vectors to spread Zika virus in China and that *Ae. aegypti* mosquitoes were better vectors than *Ae. albopictus* mosquitoes because transmission rate was higher and extrinsic incubation period was shorter for the former. The strong vector competence of *Ae. aegypti* mosquitoes could be associated with Zika virus rapid reproduction in the midgut during dpi 0–4, which enabled the viral particles to easily overcome the midgut barrier and be released into the hemolymph cavity and invade the salivary gland (*32*). Our findings are consistent with those for *Ae. aegypti* mosquitoes from Singapore and Italy (*12**,**17*). Although the distribution of *Ae. aegypti* mosquitoes is very limited in southern China, ranging from latitude 22°N to 25°N (*33*), the higher susceptibility of *Ae. aegypti* mosquitoes for Zika virus required the authorities in China to pay close attention to local epidemics of Zika virus in these regions.

Under the same experimental conditions, the whole-mosquito infection rates and midgut infection rates for *Ae. albopictus* and *Ae. aegypti* mosquitoes were similar, but the replication of Zika virus in midgut was slower for *Ae. albopictus* mosquitoes. The dissemination and transmission of Asian genotype Zika virus by *Ae. albopictus* mosquitoes in China started on 7 and 10 dpi, respectively, which indicated lower vector competence than that for *Ae. albopictus* mosquitoes from Singapore infected with East African genotype Zika virus from Uganda but higher than that for *Ae. albopictus* mosquitoes from the Americas infected with Asian genotype Zika virus from New Caledonia (*13**,**34*). Although the extrinsic incubation period was longer for *Ae. albopictus* than for *Ae. aegypti* mosquitoes, *Ae. albopictus* mosquitoes are widely distributed in China, especially in Guangdong Province, where dengue was often epidemic (*35*). Moreover, *Ae. albopictus* mosquito density and survival time has increased with urbanization (*36**,**37*). Taken together, these findings indicate that *Ae. albopictus* mosquitoes can potentially become the primary vector for Zika virus in China and need attention in the vector control strategy.

*Cx. quinquefasciatus* are common blood-sucking mosquitoes in China, especially in southern cities, and are the vector of Western equine encephalitis virus (*38*). However, in this study, at dpi 0, all *Cx. quinquefasciatus* mosquitoes had ingested the virus, but the infection rate and Zika virus copies gradually decreased and no virus was detected in any tissues after dpi 7. The few positive midgut samples before dpi 7 could have resulted from an undigested blood meal because *Cx. quinquefasciatus* mosquitoes are larger and might take more blood than *Aedes* mosquitoes. Our results illustrate that *Cx. quinquefasciatus* mosquitoes in China are not able to transmit Zika virus, a finding that is consistent with the Zika virus susceptibility of *Cx. pipiens* mosquitoes from Iowa, USA, and *Cx. quinquefasciatus* mosquitoes from Rio de Janeiro, Brazil (*15**,**39*). However, our results contradict those of Guo et al., which indicated that *Cx. p. quinquefasciatus* mosquitoes are potential Zika virus vectors in China (*40*). These contradictory results might come from different experimental conditions, virus strains, or mosquito species and need more study.

In our study, Zika virus from C6/36 cells or infected mosquitoes was sensitively and specifically identified by qRT-PCR. We used qRT-PCR to detect virus copies because the Zika virus strain isolated from the patient who imported the virus into China can infect C6/36, Aag2, and Vero cells but did not show obvious cytopathic effect, which could be associated with the patient’s mild clinical signs. Furthermore, previous research proved that the viral copies calculated by qPCR were consistent with the PFU detected by plaque assay (*41*). Although passage of the Zika virus we used in C6/36 cells was relatively low, the preliminary result demonstrated the highest virus reproduction in C6/36 cells compared with Aag2 and Vero cells.

In conclusion, our findings indicate that in China, *Ae. aegypti* and *Ae. albopictus* mosquitoes are susceptible to Zika virus, whereas *Cx. quinquefasciatus* mosquitoes are not able to transmit the imported Zika virus. Comparatively, the vector competence of *Ae. albopictus* mosquitoes is inferior to that of *Ae. aegypti* mosquitoes, but considering their wide distribution, *Ae. albopictus* mosquitoes might become the primary vector for Zika virus in China. These updated findings can be used for Zika virus disease prevention and vector control strategy.
